# *In-vitro/In-vivo* comparison of leuprolide acetate release from an in-situ forming plga system

**DOI:** 10.1186/2008-2231-21-57

**Published:** 2013-07-15

**Authors:** Roya Mashayekhi, Hamid Mobedi, Jamal Najafi, Marjan Enayati

**Affiliations:** 1 Faculty of Biomedical Engineering Department, Islamic Azad University, Science and Research Branch, Tehran, Iran; 2 Iran Polymer and Petrochemical Institute, P.O. Box:14185/458, Tehran, Iran; 3 Center of Veterinary, Tehran, Iran; 4 Pharmaziezentrum, Althanstrasse 14, Universität Wien, Raum 2D177, Wien, Austria

**Keywords:** Drug delivery system, In-vitro, In-vivo, Biodegradable polymer, Rat, Poly (lactide-co-glycolide) (PLGA)

## Abstract

A poly (lactide-co-glycolide) (PLGA) implant was used to control the release profile of leuprolide acetate (LA) drug. The system is an in-situ polymeric precipitation system. And the formulation consisted of PLGA polymer, LA drug and N-methyl-2-pyrrolidon solvent with no additives. First, the formulation was injected into PBS solution for in-vitro studies and then it was administered to the animal models (female rats) for in-vivo release studies. The release profiles of leuprolide acetate were measured by UV spectrophotometry for a period of 28 days. The initial burst release of LA was 14% in in-vitro whereas it was 7% in in-vivo. In-vitro and in-vivo release profiles of LA had similar trends after 72 hours. However, the rate of LA release was slower in-vivo. This might be attributed to the limited diffusion process of solvent and the drug molecules. This could be due to presence of an additional pressure caused by the surrounding tissue and also the presence of small amount of water between cells in the subcutaneous site. Cross-section and surface of the implants were studied via scanning electron microscopy. Morphology of both in-vitro and in-vivo implants confirmed the release behaviours. No toxicity effects were reported in the histopathological assay. Furthermore, the pharmacological analysis showed more inactive ovaries due to release of LA.

## Introduction

An injectable in-situ polymeric system forms a solid or semisolid drug reservoir upon the parenteral injection of a drug solution [1]. This is due to the permeation of water molecules into the implant, solvent removal process and coagulation of the polymer after injection [2,3]. These systems have many advantages such as; ease of administration, having less complicated fabrication, increasing effective pharmacological duration, preserving the effectiveness of short lifetime drugs and reducing the need for repeated daily injection. However, they suffer from one major disadvantage, which is their high burst release defined as the amount of drug released during the first 24 hours [4,5]. An undesirable high burst release may exhaust the loaded drug from the implants too rapidly and cause severe toxicity problems. It can also lead to an overall shorter drug release duration, and subsequently less therapeutic efficiency and cost-effectiveness.

Leuprolide acetate has been utilised for the treatment of the various diseases such as: advanced prostate cancer, breast cancer, uterine fibroids, endometriosis, and Alzheimer [6]. LA is a synthetic analog of endogenous Gn-RH agonist and a non-apeptide drug. It increases the circulating levels of the luteinizing hormone (LH) and follicle-stimulating hormone (FSH) leading to a transient increase of gonadal steroids. In females, continuous administration of LA reduces the level of LH and FSH. But in males, the level of the testosterone increases right after the initial drug administration and then falls below the castrate threshold for the next 2–4 weeks. LA can be administered via parenteral and pulmonary routes [7,8]. And it can be incorporated in different types of drug carriers such as nano/micro particles, capsules and in-situ forming implants. For instance, LA was successfully loaded in PLGA/PLA microspheres as an osmotical driven implantable system [9,10].

PLGA is a FDA approved biodegradable alpha polyester. It has been used in several therapeutic applications and can be utilised in various forms of controlled release devices. For instances, it has been used as a suture mesh for a guidance around nerve defects, as a scaffold for cell proliferation [11] and as drug delivery carriers with various forms. Moreover, N-methyl-2-pyrrolidone (NMP) is a water-soluble, water-miscible, biocompatible, and inert organic solvent with low toxicity potential in animal and human studies [12].

The main objective of this study is to investigate the release profile of leuprolide acetate from a PLGA injectable implant using in-vitro and in-vivo studies. PLGA implants were prepared and were injected into phosphate buffer saline (PBS) solution for the in-vitro studies. Subsequently, the same formulations were administered subcutaneously to the rat animal model for in-vivo studies. Drug release profiles were characterised using UV spectroscopy method. Structure and morphology of the PLGA implants were investigated in-vitro and in-vivo via scanning electron microscopy. The toxicity effects of the implants on the living tissues were investigated by a histopathological assay on sexual and non-sexual organs. Furthermore, the pharmacological analysis was conducted to study the influence of the LA release on the ovaries and the uterine of the animal models within 28 days.

## Materials and methods

### Materials

Poly (lactide-co-glycolide) (50:50), Resomer® RG 504, (Mw=48,000 Da) as a biodegradable copolymer, was obtained from Boehringer Inc. (Ingelheim, Germany) for sterilization. Leuprolide acetate (Mw 1209 Da) was supplied by Bachem Inc. (Bubendorf, Switzerland). Analytical grade N-methyl-2-pyrrolidon was purchased from Merck (Riedel-deHaen Company, German).

### Methods

#### Sample preparation

The RG 504 PLGA polymer and NMP as a solvent were used. First, the polymer was mixed with NMP in the specific ratio of PLGA (28 wt %) to NMP (69 wt %) by a glass mixer. Subsequently, the mixture was placed on a continuous shaker in order to obtain clear solutions after 1 hour. The PLGA solutions were gamma irradiated (dose of 25kGy). Then 3 wt % leuprolide acetate was dissolved in the polymer solution [13]. The sample preparation was performed at ambient temperature (23°C ± 3°C).

#### In-vitro studies

0.5 g of the formulations were injected into 15 ml PBS (0.2 M, pH 7.4) through a 20 gauge needle and were stored at 37°C in an incubator. 15 ml PBS released media was taken and replaced at the determined time points (0, 1, 2, 3, 7, 14, 18, 21, 25, 28 days). The concentration of the leuprolide acetate was calculated by UV spectrophotometer.

#### In-vivo studies

Female Sprauge-Dawley rats (8 weeks old, 150±5 g) were used. The formulation was injected subcutaneously on the back using 16 gauge needle. The animal models were divided into 3 groups (Group A, B and C). Group A (n=21) were administered with 100 (μg/kg/day) of LA [14], and Group B (n=21), received the formulation without drug 10 (μg/kg/day) of formulation. Group C (n=3) was the control group and they were sacrificed in the first day. This point is important that the research on animals was acted in pathology department in "Iran Veterinary Organization" with ethical approval (ISO 17025) from TUV (Germany).

#### Determination of leuprolide acetate (LA)

UV spectrophotometer (CECIL 1000 SERIES, England) was used for in-vitro and in-vivo release studies. In in-vitro release studies, the amounts of drug released in PBS were measured at discrete time intervals. For in-vivo release studies, the remainder PLGA implants were first dissolved in 10 ml NMP. Then, the amounts of the leuprolide acetate in the implants were calculated. Samples without LA were also monitored so that any contributions to the measured absorbance from the polymer could be evaluated.

The UV-spectroscopy method was carried out at wavelength of 279 nm. The developed method was validated with respect to linearity, accuracy (recovery), precision and specificity in accordance with ICH (International Conference on Harmonisation) requirements for assay method determination [15].

Specificity was investigated by determining the blank dissolution media and the dissolution media containing LA and any other potential excipients by UV, then spiking the drug substance and examining any interference between peaks. To determine the linearity and range of the assay method, standard solutions of LA having known concentrations were used; these standards have been analysed with the UV method as described. For each concentration, 3 runs have been performed. Accuracy was evaluated by analysing synthetic mixtures spiked with known quantities of drugs. Linearity was studied in the range of 1–25 μg/ml of the analyte content. The calibration curve obtained had a R2 value of 0.998, an intercept of 0.004 and a slope of 0.007; the intervals at 95% confidence level, calculated for the intercept included the zero value. Accuracy was determined at three levels of content in placebo. Recovery ranged from 98 to 103.5 with a mean value of 101.3 a RSD % of 0.85 and finally in the specificity term we have not seen any interference between API and other excipients. Thus the method showed an acceptable degree of linearity, accuracy and precision according to the validation requirements and can be considered valid.

#### Scanning electron microscopy (SEM)

The surface morphologies and the cross-sections of the PLGA implants were studies by SEM (Cambridge S360, England) at predetermined times (24 h, 3, 7, and 14 days). Samples were frozen and mounted on metal stubs with double-sided carbon tape and coated with gold (30 mA for 5 min) by a sputter coater (E 5200 AUTO, BioRAD Polaron Devision, England). The morphologies of the in-vivo samples after the day 14 were not studied because of significant degradation of the implants.

#### Pharmacological and histopathological analysis

The animal models were scarified at the defined time points. Their non-sexual organs (lung, liver, kidney) and sexual organs (four layers of uterine and ovaries) were fixed with 10% neutralised formalin. The paraffin method was utilised for preparation of paraffin blocks and sections were cut and mounted on microscope slides. The sections were stained by hematocyline and eosin for histological investigation and these were studied using a light microscope.

#### Statistical analysis

The in-vivo and in-vitro release data were analysed statistically, with ANOVA using Minitab 14.

## Results and discussions

### In-vitro studies

**Figure 1:**
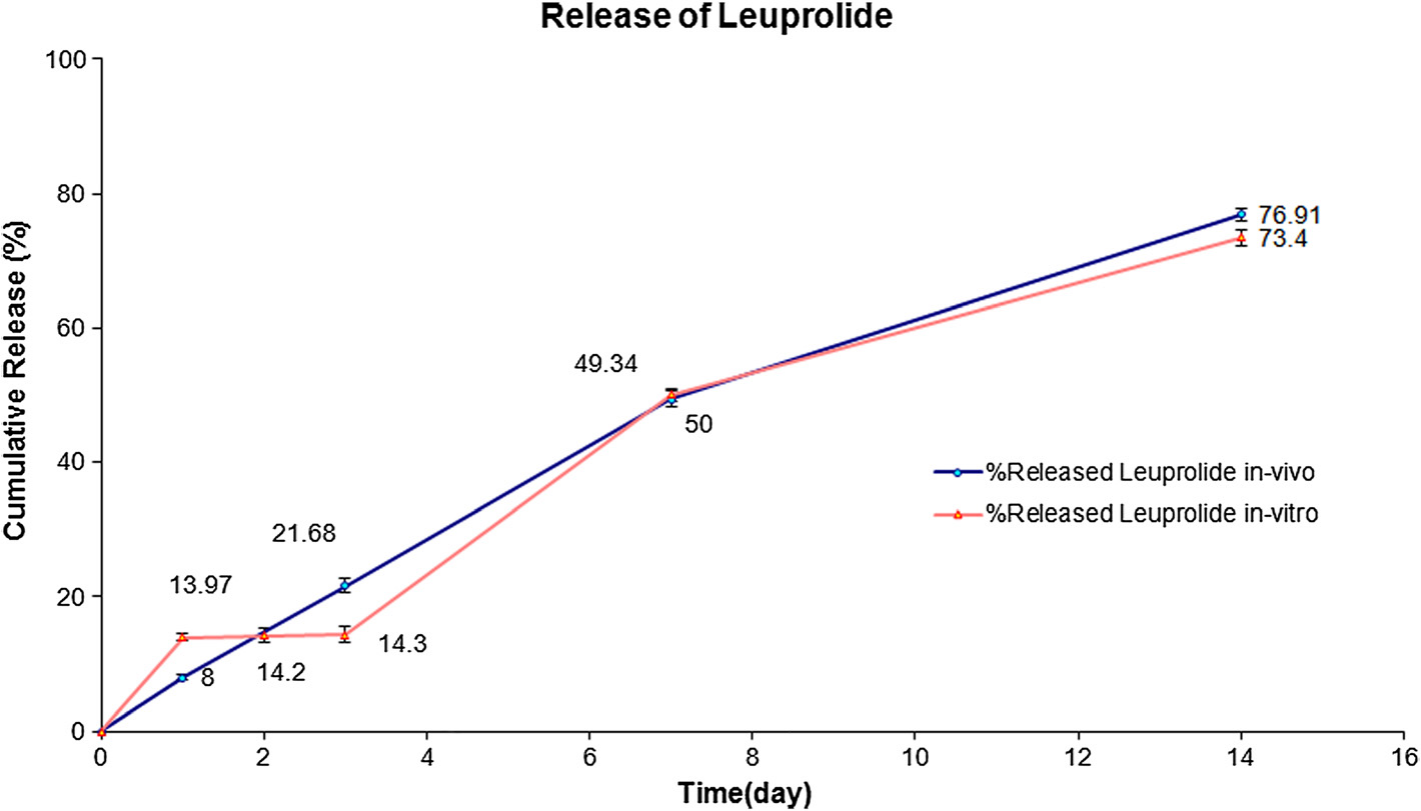
A comparison of the in-vitro and in-vivo cumulative release of LA from PLGA (50:50) systems (Mean±S.D., n=3).

**Figure 2:**
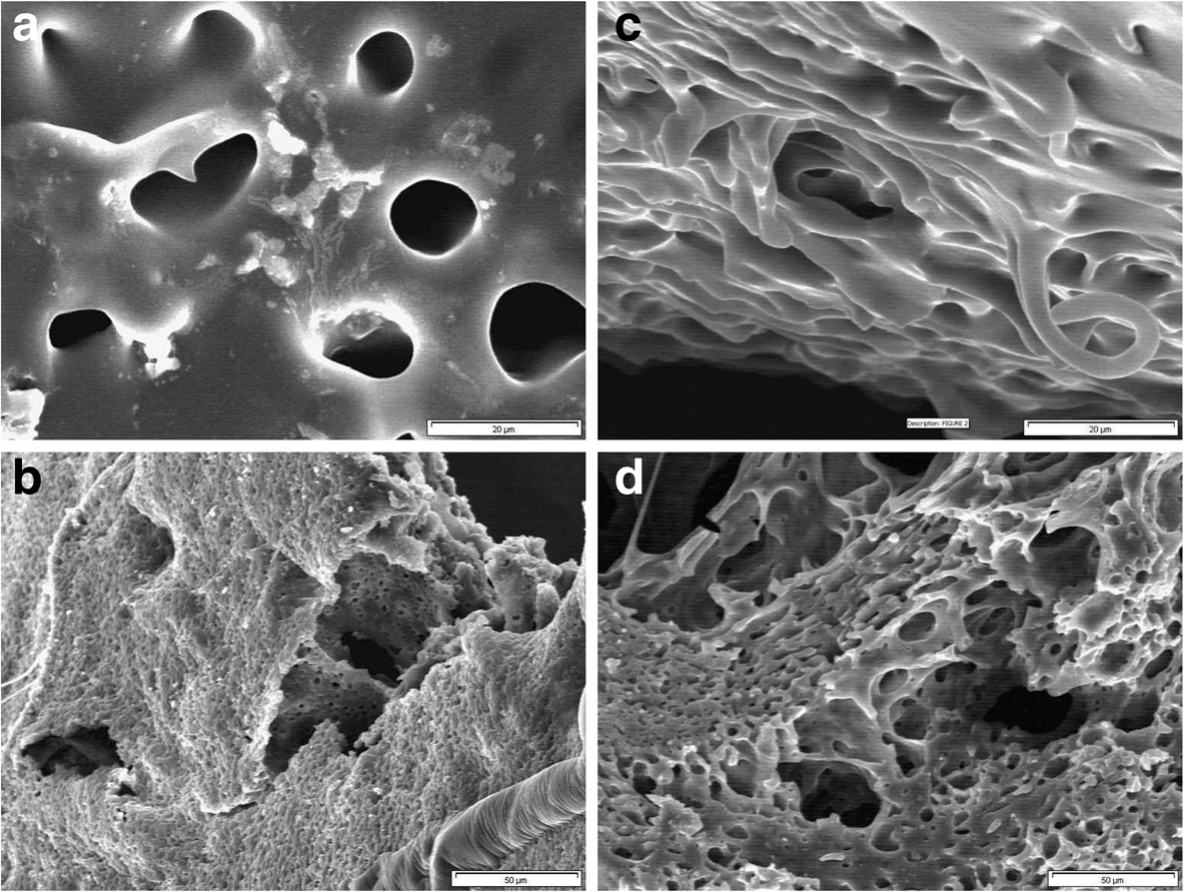
SEM images of surfaces and cross sections of the in-situ formed implants of PLGA/NMP/LA in the in-vitro environment: surface morphology after (a) 24 hours, (c) 7 days (1500x), and cross-section structure after: (b) 24 hours, (d) 3 days (500x).

Release profile of the PLGA implant is shown in Figure 1. It was observed that 14% of the drug was released initially in the first 24 hours. This initial fast release phase may be attributed to the porous surface structure and interconnected channels [16] which were observed in the SEM micrographs (Figure 2). These types of structures have been observed in previous studies utilising injectable in-situ forming PLGA implants [17].

**Figure 3:**
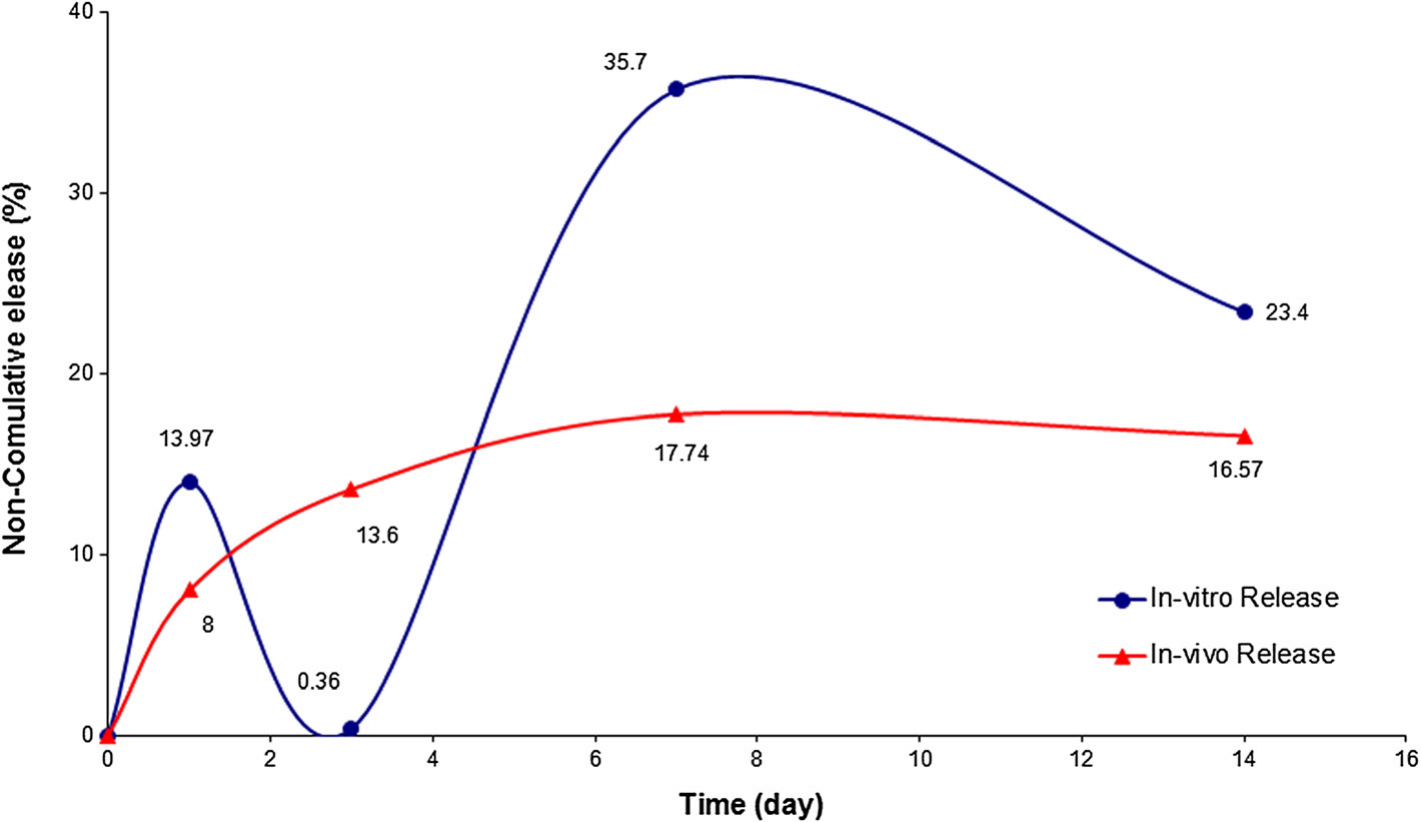
Non-cumulative in-vitro and in-vivo release profiles of the LA from PLGA implants (Mean± S.D., n=3).

The release profile was followed by a slow release phase with the rate of 1% over the next 3 days (Figure 3). These results are confirmed in the SEM micrographs (Figure 2). The degradation of the implant was not noticeable in this stage. The surface was smooth and had much less interconnected channels. However, the diameters of these channels were larger than before. The rate of the drug release was increased significantly after 72 hours. This fast release trend was maintained till day 7 and more than 50% of the loaded drug was released within this period of time (Figure 1). The surface structure of the implant showed more pores. Also, the cross-section of the implant had large tear–like structure (Figure 2) that may cause the fast release of the therapeutic agent [18]. The rate of the drug release was reduced afterward and in this final stage only 23.4 wt. % of the LA was released (Figure 3). The surface and cross-section morphologies of the prepared implants showed relatively larger interconnected channels with more pores on the surface of the implants. The PLGA implants were degraded in 28 days. And the number and size of the porosities were increased as a function of time (Figure 2).

PLGA degrades by means of hydrolysis including surface hydrolysis, bulk hydrolysis, and bulk catalysed hydrolysis. The inwards diffusion of the aqueous phase causes degradation of the polymer chains which in turn facilitates the diffusion of the entrapped drug. The biodegradation products of PLGA (lactic and glycolic acid) are common body metabolites, which are non-toxic, non-immunogenic, non-carcinogenic, and non-teratogenic. However, the products of polymer degradation can be trapped in the internal structure, which can change the localised pH within the polymer matrix. This phenomenon accelerates the polymer degradation due to autocatalysis [19].

Furthermore, PLGA erosion was determined by monitoring the pH changes and lactic acid formation as a reaction of the degradation. There was a lag-phase in formation of lactic acid in the first 4 days. The pH of the release medium was 7 and the drug release rate was not noticeable at this stage. The pH of the environment decreased afterward due to the degradation of the PLGA implant and the presence of lactic acid in the release medium [20].

In this study, the mechanism of the LA release had 4 stages: (i) diffusion of drug through the polymer; (ii) surface erosion of the polymer and continuous release of the physically entrapped drug; (iii) cleavage of the covalent bonds between the polymer bulks followed by the drug diffusion; and (iv) slow diffusion of LA through the polymer and degradation of the polymer until drug depletion.

### In-vivo studies

**Figure 4:**
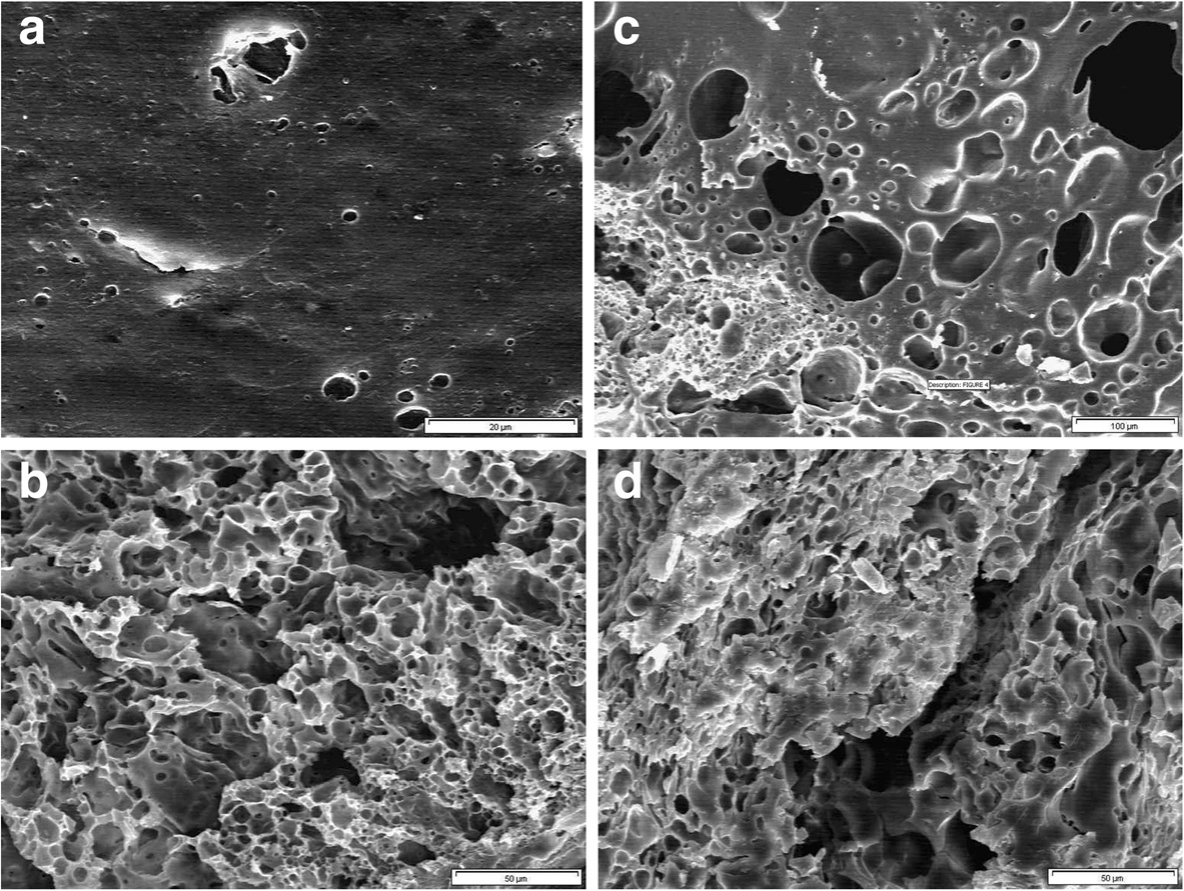
Surface and cross-section of the drug loaded PLGA implants in the in-vivo environment: surface morphology after (a) 24 hours, (c) 7 days (1500x), and cross-section structure after (b) 24 hours and (d) 3 day (500x).

The in-vivo release profile of the formulation is shown in Figure 1. It was observed that only 8% of the drug was released within the first 24 hours. This initial release was 6% less than the initial release in the in-vitro study. The surface and cross-section morphologies of the implant were also studied (Figure 4). The implant had a smooth surface whereas, many small interconnected channels were observed in the cross-section in the first 24 hours. Overall, the surfaces and the cross-sections of the implants were similar in both in-vitro and in-vivo studies. After the initial release phase, 21.6% and 49% of the drug were released in the next 3 and 7 days, respectively. SEM micrographs confirmed the in-vivo release results (Figure 4). The rate of degradation was increased significantly and more pores were observed on the surface structures of the implants. The diameters of the interconnected channels were increased throughout the bulk of the implant which results to the formation of tear-like structures. In the last stage of the release profile (from day 7 to day 14), the release rate for the sample increased significantly as degradative hydrolysis of the polymer implant took place and the central region, which would be expected to contain a higher concentration of drug, was exposed.

The morphology and structure of a blank PLGA implant (with no drug) was also studied. The SEM micrographs were similar to the SEM micrographs of the implant loaded with LA in terms of the degree of their porosities and their internal structures. These similarities were observed throughout the 28 days of the studies, except from day 3. The surface of the implant had much less pores during this period in the blank implant.

### Histopathology study

All implants used in human body should be biocompatible and non-toxic. In this study, it was observed that all tissue samples had normal structures with no necrotic tissues. And the implant does not possess any toxicity effects on the living tissues and organs including the tissues at the injection site and sexual/non-sexual organs.

### Pharmacology study

**Figure 5:**
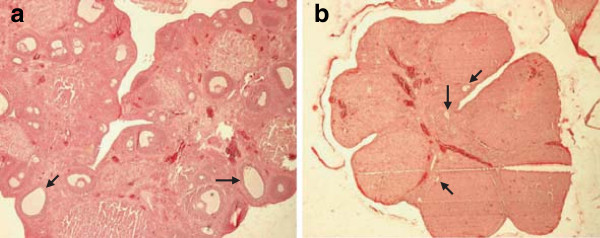
Histology images of (a) inactive ovary (control group), and (b) active ovary (injected LA formulation group) (Mean ± S.D., n=3).

Pharmacological studies showed that all the animal models, injected with the PLGA formulations with LA drug, had inactive ovaries in the first 28 days. Whereas, the ovaries of the animals injected with the formulation without the drug were completely active. These results showed that LA suppressed gonadotropin receptors effectively and stopped the ovaries functioning in the first 24 hours (Figure 5). Big white spheres in Figure 5a show activity of ovaries bacause absent of LA and in Figure 5b small white spheres showed inactivity of ovaries with the release of LA.

### Comparison of in-vitro and in-vivo results

**Figure 6:**
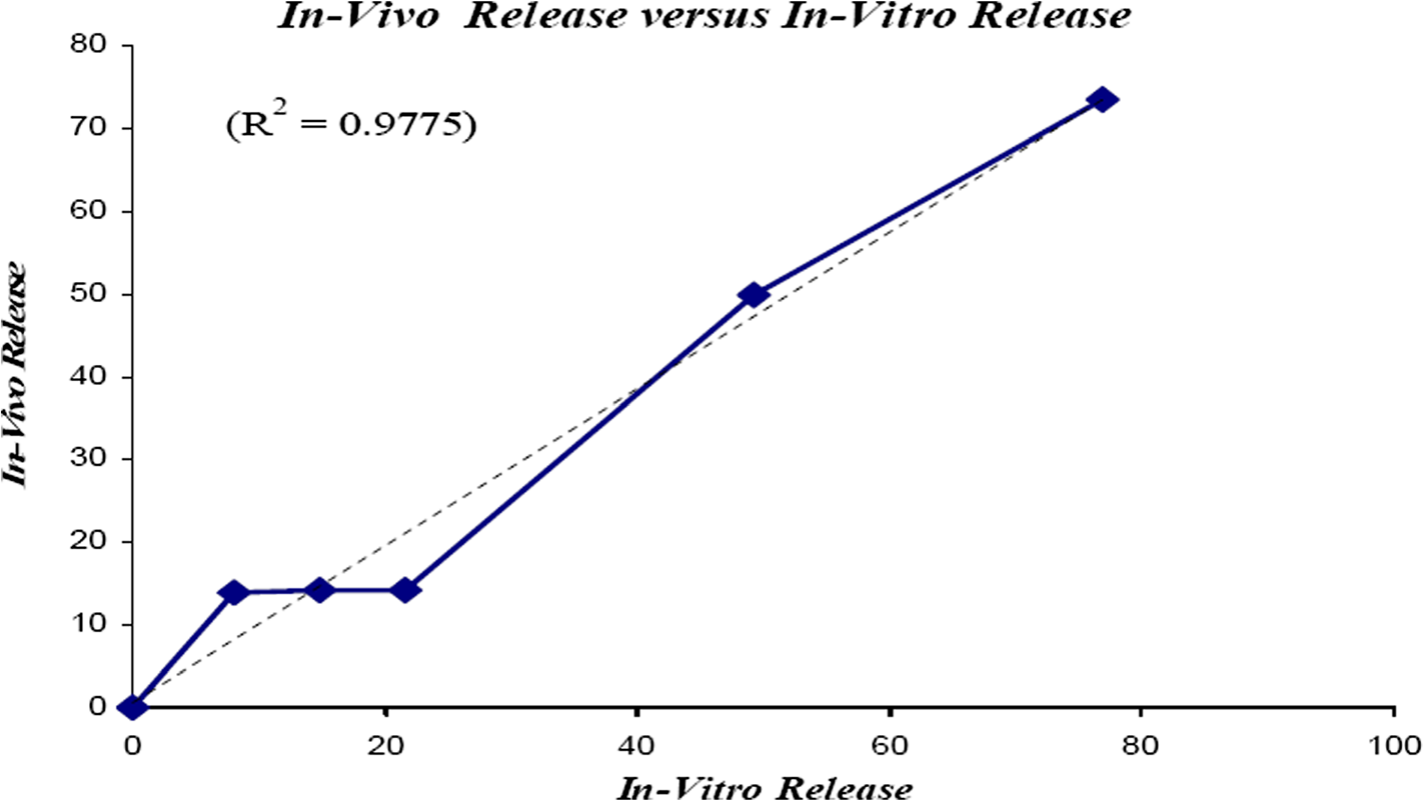
In-vivo release of LA vs. in-vitro release.

The main objective of this study was to evaluate the release profiles of the injectable PLGA implants in both in-vitro and in-vivo environments. The release results were compared and analysed statistically via Minitab 14 software. The amount of released leuprolide in-vitro and in-vivo are shown in Table 1. The drug release rates were 6% slower in the first 24 and 72 hours in the in-vivo environments. This could be due to the presence of an additional pressure caused by the surrounding tissue and also presence of small amounts of water between cells in the subcutaneous site. Afterwards, the differences between the release rates in the two environments were reduced and similar trends in release profiles were observed after day 7. The in-vivo release rate vs. in-vitro release rate graph was constructed (Figure 6). R2 of the graph was 0.9775 suggesting that the kinetics of the release profile was linear.

**Table 1:** The amount of leuprolide acetate released in in-vitro and in-vivo environments

	***In vitro***					***In vivo***				
	**Day 1**	**Day 2**	**Day 3**	**Day 8**	**Day 14**	**Day 1**	**Day 2**	**Day 3**	**Day 8**	**Day 14**
***Test 1***	13	13.1	13.2	49.9	75.2	8.1	13.1	20.1	48	76.7
***Test 2***	14.7	15.1	15.3	47	68.1	9.1	13.9	22.1	51	74.8
***Test 3***	14.2	14.6	14.7	51.2	76.9	6.8	15.6	22.9	51.2	79.2
***Mean***	14.0	14.3	14.4	49.4	73.4	8.0	14.2	21.7	50.1	76.9

## Conclusion

The release profiles of LA from injectable in-situ forming PLGA implants were studied in in-vitro and in-vivo environments. The release profiles had four phases in the in-vitro study. It started with a burst release phase where 14% of the loaded drug was released in the first 24 hours. The rate of the drug release in the initial phase was lower in the in-vivo environment and the amount of release drug was only 8% in the first 24 hours. However, the release profiles had similar trends after day 3 in both in-vitro and in-vivo environments. Histopathology studies confirmed that the implants were biocompatible and no toxicity effects were observed on the various living tissues and organs. Pharmacology studies revealed that the LA loaded implants were effective in suppressing the gonadotropin receptors and halting the ovaries function.

## Competing interests

We declare that we have no competing interests.

## Authors’ contributions

RM, HM, and ME in studies and experimental laboratories, also JN carried out histopathological. All authors read and approved the final manuscript.

## References

[B1] Hammer KS Boisclair J Schuetz H Petersen H Goepferich A Biocompatibility of an Injectable in-situ Forming Depot for Peptide Delivery Pharm. Sci 2010 99 4390 4399 10.1002/jps.22149 20665506

[B2] Fogueri LR Singh S Smart Polymers for Controlled Delivery of Proteins and Peptides A Review of Patents 2009 3 7 40 48 10.2174/187221109787158300 19149728

[B3] Kranz H Brazeau GA Napaporn J Martin RL Millard W Bodmeier R Myotoxicity studies of injectable biodegradable in-situ forming drug delivery system Int J Pharm 2001 212 11 10.1016/S0378-5173(00)00568-8 11165816

[B4] Bodmeier S López Esguerra V Körber M Dong WY Stability of poly (D,L-lactide-co-glycolide) and leuprolide acetate in in-situ forming drug delivery systems J.C.R 2006 115 2 158 167 10.1016/j.jconrel.2006.07.013 16963145

[B5] Dunn RL Garrett JS Ravivarapu H Chandrashekar BL Polymeric delivery formulations of Leuprolide with improved efficacy US Patent 2004 6 773,714

[B6] Okada H Doken Y Ogawa Y Persistent Suppression of the Pituitary-Gonadal System in Female Rats by Three-Month Depot Injectable Microspheres of Leuprorelin Acetate J Pharm Sci 1996 85 10 1045 10.1021/js960123a 8897268

[B7] Hatano T Incidence of bone fracture in patients receiving luteinizing hormone-releasing hormone agonists for prostate cancer BJU Int 2000 86 449 452 10.1046/j.1464-410X.2000.00774.x 10971270

[B8] Eligard SO leuprolide acetate in a novel sustained-release delivery system Urology 2003 61 2Suppl 1 25 31 12667884 10.1016/s0090-4295(02)02396-8

[B9] Bari H Inter A, prolonged release parenteral drug delivery system Inter. J Pharm. Sci. Rev. Res 2010 3 1 1 11

[B10] Zare M Mobedi H Barzin J Mivehchi H Jamshidi A Mashayekhi R Effect of Additives on Release Profile of Leuprolide Acetate in an In-situ Forming Controlled-Release System: In-vitro Study J. Appl. Poly. Sci 2008 107 3781 3787 10.1002/app.27520

[B11] Krogman NR Singh A Nair LS Laurencin CT Allcock HR Biomacromolecules 2007 8 13106 13125

[B12] Dunn RL Garrett JS Ravivarapu H Chandrashekar B Polymeric delivery formulations of leuprolide with improved efficacy US Patent 2003 656 5874

[B13] Strickley RG Solubilizing excipients in oral and injectable formulations Pharm Res 2001 21 201 230 15032302 10.1023/b:pham.0000016235.32639.23

[B14] Okada H Inoue Y Heya T Ueno H Ogawa Y Toguchi H Pharmacokinetics of once-month injectable microspheres of leuprolide acetate Pharm Res 1991 8 6 787 791 10.1023/A:1015818504906 1905810

[B15] Guideline, I.C.H.H.T **Validation of analytical procedures: Methodology** ICH 1996

[B16] Luan X Bodmeier R Influence of the poly (lactide-co-glycolide) type on the Leuprolide release from in-situ forming microparticle systems J.C.R 2006 110 266 272 10.1016/j.jconrel.2005.10.005 16300851

[B17] Bakhshi R Vasheghani-Farahani E Mobedi H Jamshidi A Khakpour M The effect of additives on naltrexone hydrochloride release and solvent removal rate from an injectable in-situ forming PLGA implant Poly. Adv. Tech 2006 17 354 359 10.1002/pat.717

[B18] Luan X Bodmeier R Modification of the tri-phasic release pattern of Leuprolide Acetate-loaded poly (lactide-co-glycolide) microparticles Euro. J. Pharm. Biopharm 2006 63 205 214 10.1016/j.ejpb.2005.12.010 16621485

[B19] Khang G Kyuang JE Rhee JM Controlled release of nerve growth factor from sandwiched poly (D, L-lactide-co-glycolide) films for the application in neural tissue engineering Macromol Res 2003 11 5 334 340 10.1007/BF03218373

[B20] Astaneh R Moghimi HR Erfan M Mobedi H Formulation of an injectable implant for peptide delivery and mechanistic study of the effect of polymer molecular weight on its release behavior DARU 2006 14 2 65 70

